# High Diversity and Transmission Dynamics of HIV-1 Non-C Subtypes in Bangladesh

**DOI:** 10.3390/tropicalmed8090451

**Published:** 2023-09-17

**Authors:** Md. Safiullah Sarker, Rubiyat Jahan

**Affiliations:** 1 Virology Laboratory, International Centre for Diarrhoeal Disease Research, Bangladesh (icddr,b), Dhaka 1212, Bangladesh; 2 School of Business and Economics, North South University, Dhaka 1229, Bangladesh; rubiyat.sarker@northsouth.edu

**Keywords:** HIV, non-C subtype, recombinant, migrant workers, Bangladesh

## Abstract

Genetic diversity and molecular epidemiology of HIV are directly relevant to HIV transmission. We report here the genetic diversity and transmission dynamics of non-C subtypes of HIV-1 strains detected in Bangladeshi key populations. Sequence analysis of *gag* gene revealed four subtypes A1, B, D, G, and nine CRFs (01_AE, 02_AG, 09_cpx, 10_CD, 15_AE/B, 13_cpx, 14_BG, 22_01_A1, and 25_AGU). Most of these non-C strains were detected in returnee migrant workers from different parts of the world. Phylogenetic analysis showed that the Bangladeshi HIV-1 strains detected in migrant workers and their wives and local sex workers shared common ancestries. The identification of the multiple subtypes indicates high diversity of non-C HIV-1 variants circulating in Bangladesh which might have been imported by migrant workers from multiple geographical areas.

## 1. Introduction

During the last World AIDS Day on 1 December 2022, there were an estimated 39 million individuals across the globe living with Human Immunodeficiency Virus (HIV). Out of this number, approximately 37.5 million were adults, while 1.5 million were children under the age of 15. It is noteworthy that women and girls accounted for 53% of these cases [1]. In some countries, people living with HIV often face higher overall mortality rates when compared to both the general population and individuals who do not have HIV [2]. Within the South East Asia region, an approximate 3.8 million individuals are currently residing with HIV, constituting approximately 10 percent of the global HIV burden. In the year 2021, an estimated 82,000 individuals within this region succumbed to AIDS-related causes, contributing to over 12 percent of the global AIDS problem. These statistics underscore the substantial impact of HIV/AIDS within the South East Asia region, emphasizing the urgent need for intensified efforts in prevention, treatment, and support initiatives to curtail the progression of the disease and to reduce its associated mortality rates [3].

As per the information from UNAIDS, the Joint United Nations Programme on HIV/AIDS, Bangladesh has documented over 14,000 cases of individuals afflicted with AIDS, which accounts for 0.01 percent of the total population. However, the current count of patients receiving treatment stands at 8000. Up until 30 November 2021, Bangladesh recorded a cumulative total of 1588 AIDS-related deaths. Meanwhile, no further records on this matter have been obtained [4].

Upon analyzing the data collected by the Directorate General of Health Services (DGHS), it becomes evident that the incidence of AIDS cases is progressively rising within the country. The majority of the recent HIV infections are traced back to expatriates or their family members. While cases had been identified in four distinct population groups earlier, infections among the general population have seen a rise over the past year [4].

### 1.1. Historical Overview of HIV/AIDS in Bangladesh

The initial instance of HIV appearing in Bangladesh was documented in 1989. Since then, annual reports have indicated the identification of 10, 20, 100, or 200 new cases per year. The year 2018 saw 869 new cases being recorded. Bangladesh marked its first HIV-infected patient’s demise in the year 2000.

According to data up until 30 November 2021, Bangladesh conducted HIV tests on a total of 628,312 individuals during the previous year. Furthermore, 662,757 individuals underwent blood screening procedures. Throughout 2021, Bangladesh identified an additional 729 cases of HIV infection. Among these cases, 188 were Rohingya refugees originating from Myanmar. In tandem with them, the cumulative count of potential HIV-infected individuals within the country reached 14,000.

During the same timeframe, the distribution of the new cases was as follows: 186 (26%) within the general population, 188 (26%) among Rohingyan refugees, 144 (20%) among expatriates and their family members, 81 (8%) among injecting drug users, 17 (2%) among female sex workers, 67 (9%) among homosexuals, 53 (7%) among male sex workers, and 13 (2%) among transgender individuals [4].

### 1.2. Returning Expatriates Raises Alarm and Lack of Awareness

Since 2019, a significant number of expatriates have returned to their home country due to the global COVID-19 pandemic. This trend has raised concern because research findings reveal that approximately 30% of recently diagnosed HIV patients are either returning migrants or their family members. The transmission rate of HIV is notably higher among these groups. During the same period, the government has primarily focused on managing the COVID-19 crisis and implementing home quarantine measures. As a result, there is growing anxiety that adequate AIDS testing and treatment may be neglected.

Experts note that the current level of AIDS awareness in the country is insufficient. Although it has been gradually increasing: thanks to the extensive awareness campaigns by the government. Reports from the National AIDS/STD Programme of the Directorate General of Health Services (DGHS) show that nearly half of those with HIV are unaware of their infection, and over a third of those who are aware refrain from seeking treatment. Managing HIV requires consistent medication and regular blood tests, yet the government currently lacks comprehensive data on the treatment status of these individuals, despite efforts to address this information gap initiated in late 2019.

Notably, the awareness level among previously high-risk groups like sex workers has substantially improved, and returning expatriates are also becoming more informed about HIV. Strengthening these awareness initiatives could further mitigate the risk of AIDS [4].

### 1.3. HIV Transmission in Bangladesh: Role of Migrants and Sexual Behavior

According to reports, 25–40% of new HIV infections in various Asian countries are caused by the wives and girlfriends of males who acquired the virus through paying women for sex, engaging in sexual activity with other men, or injecting drugs with non-sterile needles or syringes [5,6]. In addition, in many Asian countries, HIV has been detected among returnee migrants who acquire the infection while abroad and then transmit the virus to the local population [7,8]. A survey shows that many of the Bangladeshi migrant workers while abroad engage in behaviors that put them at risk of HIV [9]. Many married men claimed to have engaged in sexual activity with female sex workers, whether they had traveled within Bangladesh or abroad [10]. Compared to women who were not separated, more women whose husbands had departed reported having concomitant extramarital sex [10]. The data on the HIV epidemic relies on the national HIV surveillance that is being conducted since 1998 among key populations (KP) such as people who inject drugs (PWID), sex workers, males having sex with males (MSM), and transgendered people (referred to as hijra in Bangladesh). Additional information is gathered from the Voluntary Counseling and Testing (VCT) centers located in different parts of the country which are compiled annually by the Health Ministry of the Govt. of Bangladesh. HIV prevalence is very low in Bangladesh, compared to neighboring countries, India, Nepal, and Myanmar [11], and information on HIV-1 genotypes and their possible origin and transmission is limited. In our previous genotyping report with 198 HIV-1 positive strains, we showed that genotype C and associated recombinants (CRF07_BC and CRF08_BC) accounted for 68.2% of the HIV infections during 1999–2005 and were mainly restricted to PWID [12]. In contrast, non-C HIV-1 strains (31.8%) were circulating in different KPs as well as in the general population. 

In the present study, we aimed to characterize the *gag* gene (and some *env* gene) of non-C HIV strains and investigate their origins and transmission by phylogenetic analysis.

## 2. Materials and Methods

### 2.1. Sample Collection

HIV non-C subtype strains were detected from leftover blood samples collected for the national HIV surveillance among KP in Bangladesh and the VCT Unit of icddr,b between March 2002 and May 2005. These samples were collected and stored at −20 °C, as described in our previous paper [12]. All study participants provided informed, written agreement prior to blood collection, and in the instance of the one child who tested HIV positive, parental consent was also acquired. For those who could not read, the summary of the consent form was read, and for those who could not sign, a left thumbprint was taken.

### 2.2. Subtyping

Using nested polymerase chain reaction (PCR), proviral DNA was isolated from whole blood samples and amplified at the junction (p17/p24) of the *gag* gene as previously described. [12]. The amplified products were nearly 400 bp (corresponding to positions 836–1249 in the HXB2 reference strain). We also randomly amplified and sequenced seven strains for the *env* region to determine whether they were truly recombinant. For *env* region, the primer for the first round PCR was ED5/ED12 (corresponding to positions 6517–7771 in the HXB2 reference strain) and for 2nd round, it was 826/308 (corresponding to positions 6967–7382 in the HXB2 reference strain). The basic PCR conditions were as follows: initial activation for 2 min at 95 degrees Celsius, then 35 cycles of 94 degrees Celsius for 30 s, 62 degrees Celsius for 45 s, 1 min at 72 degrees Celsius, and a final extension for 5 min in a final volume of 50 µL. The secondary PCR conditions were as follows: initial activation for 2 min at 95 degrees Celsius, 35 cycles of 94 degrees Celsius for 30 s, 58 degrees Celsius for 45 s, 1 min at 72 degrees Celsius, and a final extension for 5 min in a final volume of 25 µL. The second cycle of PCR was conducted using the same reaction mixture and three microliters of the initial amplified product. Electrophoresis on a 1.5% agarose gel was used to identify the PCR products, and ethidium bromide staining was used to make them visible.

On an ABI 377 automated DNA sequencer, the amplified DNA was sequenced. Using Chromas 2.23 (Technelysium, Brisbane, QLD, Australia), the chromatogram sequencing files were examined, and SeqMan II was used to create the consensus sequences (DNASTAR, Madison, WI, USA). ClustalX1.81 was used to calculate multiple sequence alignments [13]. HIV genotyping tools (http://www.ncbi.nlm.nih.gov/projects/genotyping/formpage.cgi (accessed on 21 August 2023). were used to subtype partial sequences of the *gag* and *env* genes (at least 300 nucleotide bases). A manual phylogeny using reference strains from the Los Alamos HIV sequencing library was used to confirm all subtypes (www.hiv.lanl.gov; accessed on 21 August 2023). Finally, 349 base pair sequences of 33 *gag* genes were evaluated. Sequences at the 5′ and 3′ ends were excluded from the alignment for those that could not be aligned clearly due to length variability of the sequences. The MEGA version 6 software program was used to perform phylogenetic analyses using the neighbor-joining approach and to determine genetic distances using the nucleotide p-distance model [14].

The accession numbers JX310758 through JX310790 (for the *gag* sequence) and KF421247-KF421251 were used to submit the nucleotide sequences to GenBank, NCBI (for *env* sequence).

## 3. Results

A total of 63 HIV strains were identified as non-C subtypes, 52 of which were from clients of the VCT unit and 11 from participants in the national HIV surveillance. The mean age of the study participants was 30.7 years, which ranged from 2 to 45 years. A total of 73% were male, 24% were female, and the remaining 3% were hijra (*transgender)*. Migration history was available for 47 VCT clients (Table 1) of which 33 said that they had worked abroad; 6 were presumed to have been related to partners who had worked abroad, and 8 were non-migrant. Most of the migrants had traveled to the Middle East (UAE, Saudi Arabia, Qatar). Some of them worked in South Asia (Pakistan and India) and South East and East Asia (Malaysia, Singapore, Thailand, Vietnam, South Korea), and the United States. All migrant workers claimed to have purchased sexual services from foreign women; several of them also had numerous risk factors, such as using injectable drugs, having intercourse with other men, and receiving blood transfusions while overseas. Migration history was not available for any of the 11 surveillance participants.

Among the non-C HIV-1 strains, subtype A, G, and related recombinants were the most predominant (n = 50, 79.3%) followed by subtype B and related recombinant (n = 7, 11.1%), subtype D and related recombinant (n = 6, 9.5%). A total of three phylogenetic trees were constructed to determine their genetic relatedness among HIV-1 strains.

### 3.1. HIV Subtype A or G and Related Recombinant 

**Figure 1:**
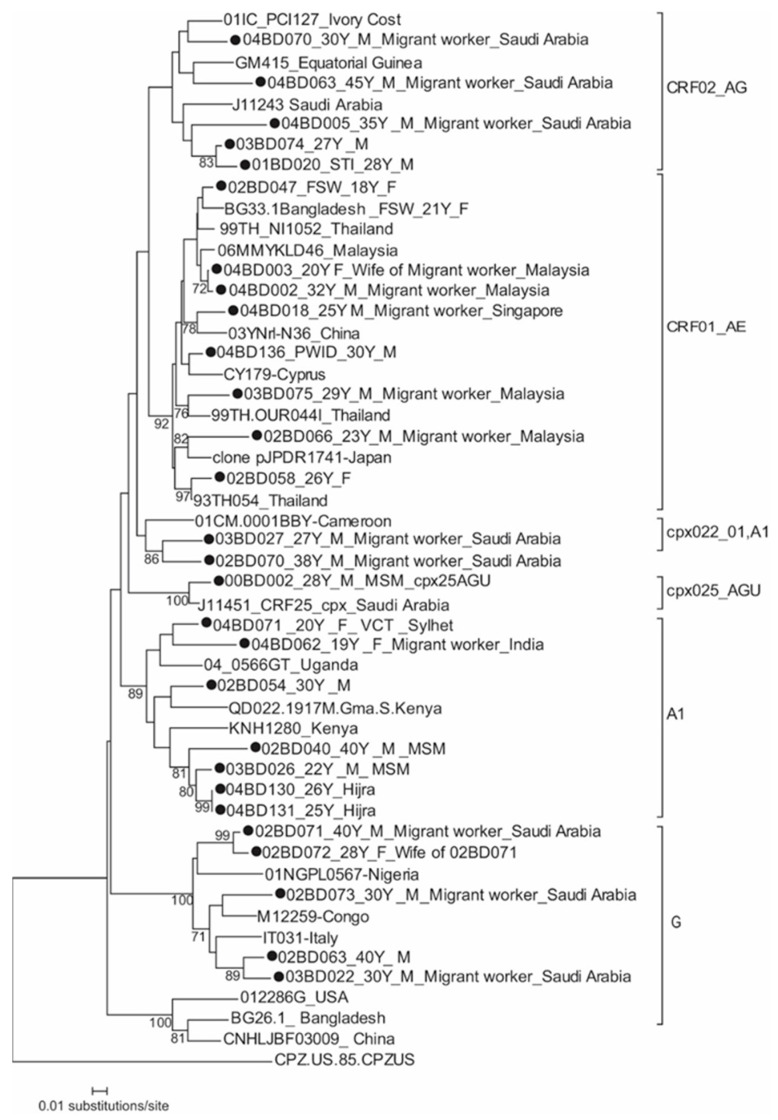
Neighbor-joining phylogenetic tree based on nucleotide sequences of the partial gag encoding gene (349 nt bases) for HIV-1 subtype A1, G, CRF01_AE, CRF02_AG, CRF22_01_A1, and CRF25_AGU. The numbers adjacent to the nodes represent the value of bootstrap support (100 replicates) for the clusters to the right of the node. Bootstrap values lower than 70% were not shown. The tree is rooted using simian immunodeficiency virus CPZ.US.85. M, male; F, female. The Bangladeshi strains are with a filled circle.

The first phylogenetic tree included HIV-1 subtypes A1, G, and their related recombinants AE, AG, and AGU (Figure 1). 

The two identical A1 strains 04BD130 and 04BD131 from hijra were closely related to MSM strains 04BD026, 02BD040, and clustered in the same branch with a Kenyan strain. A high nucleotide identity (96%) amongst the A1 strains from MSM and hijra suggests close sexual links between these groups. The other Bangladeshi A1 strains 04BD062, 02BD054, and 04BD071 clustered with strains from Africa.

Eight Bangladeshi CRF01_AE strains clustered with Asian lineage, which includes Bangladeshi female sex worker strains 02BD047 and BG33.1 as well. Five of them returned from Malaysia and Singapore and they all said that they had sex with female sex workers while abroad. The remaining strains were identified from a female sex worker, a housewife, and a male PWID. They were also similar to Asian strains (Figure 1).

Two subtype G strains (02BD071 and 02BD072 with 98.5% nucleotide identity) were identified in a married couple and clustered together with the Nigerian strain 01NGPL0567. The husband was a returnee migrant worker from Saudi Arabia who had sex with a female sex worker while working abroad. The other two subtype G strains, 02BD063 and 03BD022, were placed in a different branch with an Italian strain IT031. The fifth strain 02BD073, which was identified from a male returnee migrant worker from Saudi Arabia, clustered in separate branch distantly related to other Bangladeshi strains and was placed with a West African strain (M12259 from Congo). Bangladeshi CRF02_AG strains were closely related to each other and clustered with strains from Saudi Arabia, Equatorial Guinea, and the Ivory Coast. The other two CRF22_01_A1 strains (03BD027 and 02BD070) from two returnee male migrant workers from Saudi Arabia clustered with African strains (01CM.0001BBY from Cameroon). Only one CRF25_AGU strain was identified which was very similar to a Saudi Arabian strain.

### 3.2. HIV Subtype B and Its Related Recombinant

**Figure 2:**
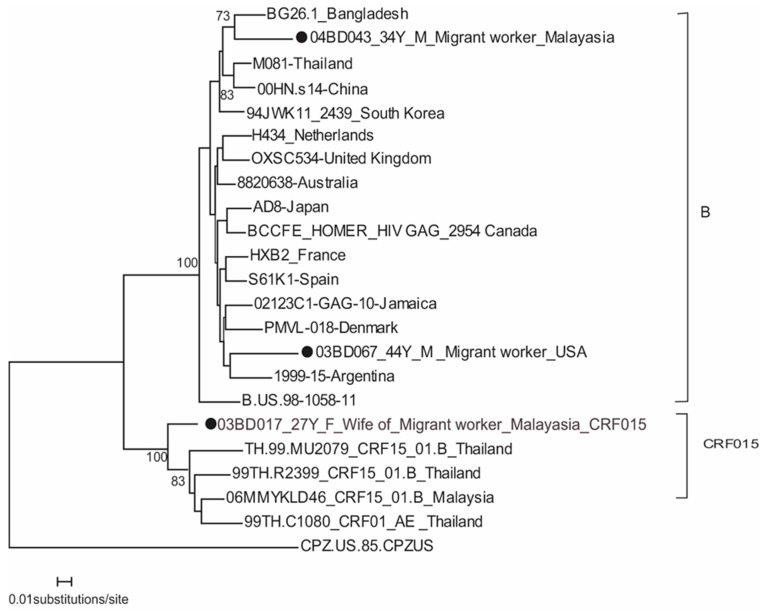
Phylogenetic tree of HIV-1 subtype B and CRF15_AE/B based on partial gag gene. The tree is rooted using simian immunodeficiency virus CPZ.US.85. M, male; F, female. The Bangladeshi strains are with a filled circle.

Figure 2 shows that two Bangladeshi subtype B strains were placed in different branches: one (02BD067) with an American and another (04BD043) with Asian strains. The single strain of CRF15_AE/B (03BD017) from a 27-year-old female who lived in Malaysia with her husband clustered with a Malaysian strain. Her husband was also infected with the virus. 

### 3.3. HIV Subtype D and Its Related Recombinant 

**Figure 3:**
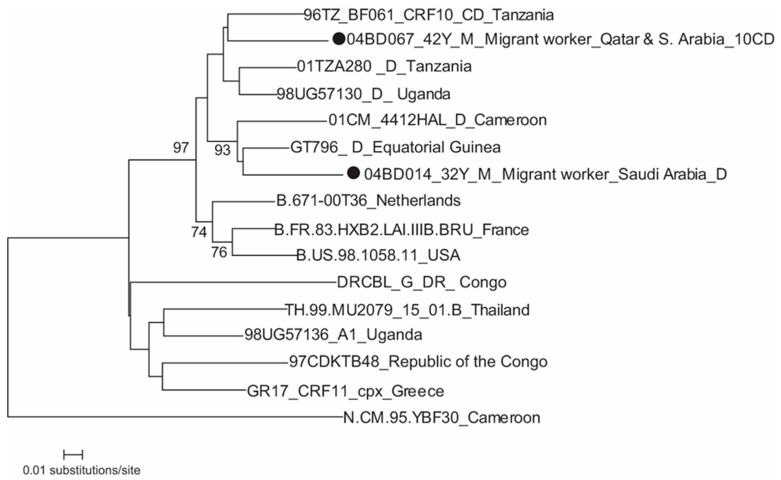
Phylogenetic tree of HIV-1 subtype D and CRF10_CD). The tree is rooted using HIV-1 group N (N.CM.95.YBF30). M, male; F, female. The Bangladeshi strains are with a filled circle.

Figure 3 shows that a subtype D strain, 04BD014, was identified from a returnee migrant worker from Saudi Arabia who had history of blood transfusion there and, as expected, clustered with two African strains (GT796 and 01CM_4412 HAL). The CRF10_CD strain 04BD067 clustered with Tanzanian strain 96TZ_BF061. This strain was identified from a male returnee migrant who worked in Qatar and Saudi Arabia for 17 years and had sex with female sex workers in Qatar. 

We were able to sequence *env* genes from five sufficiently available samples and found that the *env* subtyping results perfectly matched with the *gag* subtyping assignment (Table 2).

## 4. Discussion

This study investigated the non-C subtype of HIV-1 strains in Bangladeshi individuals and their transmission by using phylogenetic analysis along with demographic data. While considering estimated low prevalence of HIV-1 in Bangladesh and, hence, the small sample size, the level of HIV-1 strain diversity was very high. The different patterns were illustrated by the phylogenetic analysis, which revealed that the HIV-1 strains shared many branches with other strains from throughout the world. Most of those whose strains were discovered had lived for work purposes in Middle Eastern, South Asian, or South East Asian countries. The main way that HIV spreads appears to be in heterosexual participants of the study, with different risks for a couple. Between Bangladesh and the Middle East, particularly Saudi Arabia, there is a sizable labor market. Our study provides evidence that many migrant workers sampled here were primarily infected with strains while buying sex from female sex workers while in Saudi Arabia; however, these strains were genetically very close to African strains. This could be explained by the fact that most of the Saudi Arabian strains originally were imported from African countries due to close geographical vicinity and commercial interactions between these countries [15]. At the same time, similar viruses were detected in the spouses of returnee migrant workers and in Bangladeshi female sex workers, confirming that the migrant workers play a key role in spreading the viruses in the local community [16].

Genetic recombination among HIV-1 strains is a fundamental property which can be distinguished by analyzing complete genome or multiple genes (i.e., *gag*, *pol*, and *env*). The analysis of only one gene may fail to accurately characterize the recombinant subtypes. Since our findings are based on only *gag* gene sequence, we might have failed to characterize some recombinant strains accurately. However, further characterization using *env* or *pol* genes was not possible because this study used a small amount of leftover samples collected through other studies.

## 5. Conclusions

With globalization and movements of people across borders, new and diverse strains of non-C HIV subtypes, especially CRFs, are regularly being introduced in Bangladesh, which have the potential to spread within the country through the networks of non-commercial and commercial sex. The ongoing targeted intervention programs in Bangladesh, which are geared towards KPs [15,16,17,18], should include returnee migrant workers to minimize the spread of HIV in Bangladesh.

## Figures and Tables

**Table 1:** HIV-1 non-C subtypes and their genetic and demographic information.

No	Specimen ID	Sex ^1^	Age (Years)	Group	Subtype (gagp17/p24)	Migration History	Sequence Analysis			
							Most Similar Strain	Country of Origin	Accession	% nt ^16^ Identity
1	02BD054 *	M	30	VCT ^2^	A1	NA ^8^	CY178	Cyprus	EU673446	96.2
2	02BD060	M	36	VCT	A1	UAE	BCCFE_HOMER_HIV_GAG_3145	Canada	EU242202	91
3	02BD061	M	38	VCT	A1	UAE	92UG_029	Uganda	AY713407	93
4	02BD062	F	35	VCT	A1	UAE(spouse) ^9^	92UG_029	Uganda	AY713407	91
5	03BD008	M	35	VCT	A1	UAE	ML603-24.7.95	Kenya	EF164299	94
6	03BD026 *	M	22	VCT	A1	Non migrant	00KE_KER2009	Kenya	AF457053	94.6
7	05BD053	M	26	VCT	A1	UAE	HIV-1 clone 2814	Kenya	GQ432678	90
8	05BD059	F	20	VCT	A1	Non migrant	HIV-1 clone 749	Kenya	GQ430613	89
9	04BD062 *	F	19	VCT	A1	India	04_0566GT	Uganda	AY803394	93
10	04BD071 *	F	20	VCT	A1	Non migrant	CY178	Cyprus	EU673446	95.2
11	04BD130 *	H	26	Hijra ^3^	A1	NA	00KE_KER2009	Kenya	AF457053	95.2
12	04BD131 *	H	25	Hijra	A1	NA	00KE_KER2009	Kenya	AF457053	95.2
13	02BD040 *	M	40	MSM ^4^	A1	NA	00KE_KER2009	Kenya	AF457053	94
14	99BG26	M	32	PWID ^5^	B	NA	04CNLN130	China	EF122504	96
15	03BD067 *	M	44	VCT	B	USA	8820638	Australia	AY857153	93.3
16	04BD004	M	31	VCT	B	Singapore	H61	Spain	DQ854715	93
17	04BD043 *	M	34	VCT	B	Malaysia	CNHLJBF03009	China	EU131828	93.9
18	05BD051	M	26	VCT	B	Malaysia	2669	Russia	EF121245	94
19	04BD014 *	M	32	VCT	D	S.Arabia	GT796	East Guinea	AY579680	91.5
20	03BD064	M	37	VCT	D	S.Arabia	03-5432NY	Uganda	AY803362	88
21	03BD068	M	35	VCT	D	S.Arabia	CA04	Cameroon	AF247517	93
22	05BD077	F	32	VCT	D	UAE (spouse)	ELI	D.R. Congo	K03454	93
23	02BD071 *	M	40	VCT	G	S. Arabia	95LB11	Liberia	AF196694	95.5
24	02BD072 *	F	28	VCT	G	S. Arabia (spouse) ^10^	95LB11	Liberia	AF196694	98.5
25	02BD073 *	M	30	VCT	G	S. Arabia	92NG003	Nigeria	U88825	94.5
26	03BD009	F	2	VCT	G	Non migrant	89SM_145	Somalia	AY713415	92
27	03BD022 *	M	30	VCT	G	S. Arabia	95LB11	Liberia	AF196694	94.8
28	02BD063 *	M	40	VCT	G	NA	92NG003	Nigeria	U88825	93.6
29	03BD058	M	38	VCT	G	S. Arabia	92NG003	Nigeria	U88825	94
30	04BD060	M	40	VCT	G	S. Arabia	92NG003	Nigeria	U88825	91
31	05BD072	M	33	VCT	G	S. Arabia	92NG003	Nigeria	GU458665	93
32	02BD058 *	F	26	VCT	CRF01_AE	NA	93TH054	Thailand	AB220945	97.9
33	02BD059	M	30	VCT	CRF01_AE	NA	00CMNYU1162	Cameroon	EF087995	93
34	02BD066 *	M	23	VCT	CRF01_AE	Malaysia	pJPDR1741AE25	Japan	AB253640	94.8
35	02BD070 *	M	38	VCT	CRF22_01_A1	S. Arabia	00CMNYU1162	Cameroon	EF087995	93.3
36	03BD075 *	M	29	VCT	CRF01_AE	Malaysia	99TH.OUR044I	Thailand	AY358042	95.4
37	03BD021	M	28	VCT	CRF01_AE	S. Arabia	00CMNYU1162	Cameroon	EF087995	91
38	03BD027 *	M	27	VCT	CRF22_01_A1	S. Arabia	00CMNYU1162	Cameroon	EF087995	93.3
39	04BD002 *	M	31	VCT	CRF01_AE	Malaysia	06MMYKLD46	Malaysia	EF495062	98.2
40	04BD003 *	F	20	VCT	CRF01_AE	Malaysia (spouse) ^11^	06MMYKLD46	Malaysia	EF495062	98.5
41	04BD009	M	36	VCT	CRF01_AE	NA	C051M2P2350	Thailand	GU458736	93
42	04BD017	M	35	VCT	CRF01_AE	Malaysia	07LN184	China	FJ531435	94
43	04BD018 *	M	25	VCT	CRF01_AE	Singapore	93TH054	Thailand	AB220945	96
44	04BD026	M	30	VCT	CRF01_AE	Multiple countries ^12^	C051M1P579	Thailand	GU458520	95
45	05BD057	M	30	VCT	CRF01_AE	Singapore	C057M2P2430	Thailand	GU458743	94
46	04BD136 *	M	30	PWID	CRF01_AE	NA	pCM235	USA	AF259955	97
47	00BD033	F	21	FSW ^6^	CRF01_AE	NA	C116M1P2101	Thailand	GU458715	92
48	03BD112	F	24	FSW	CRF01_AE	NA	HCM309	Vietnam	AB044063	98
49	02BD047 *	F	18	FSW	CRF01_AE	NA	144.1	Australia	EF116320	97.9
50	01BD020 *	M	28	STI ^7^ patients	CRF02_AG	NA	J11243	S. Arabia	DQ375306	94.8
51	03BD074 *	M	27	VCT	CRF02_AG	NA	p03GH189AG09	Ghana	AB286861	96.5
52	04BD005 *	M	35	VCT	CRF02_AG	S. Arabia	LBV23.10	Gabon	L11779	92.1
53	04BD063 *	M	45	VCT	CRF02_AG	S. Arabia	CM52885	Cameroon	AF377954	93.5
54	04BD070 *	M	32	VCT	CRF02_AG	Multiple countries ^13^	01IC-PCI127	Ivory Cost	AJ866558	93.5
55	03BD016	M	40	VCT	CRF09_cpx (CRF02, A, U)	Multiple countries ^14^	95SN1795	Senegal	AY093603	89
56	03BD117	F	35	FSW	CRF09_cpx (CRF02, A, U)	NA	J11233	Saudi Arabia	EU697906	95
57	04BD031	F	20	VCT	CRF10_CD	S. Arabia (spouse)	96TZ-BF110	Tanzania	AF289550	89
58	04BD067 *	M	42	VCT	CRF10_CD	Multiple countries ^15^	GT796	Equatorial Guinea	AY579680	88.1
59	05BD074	M	32	VCT	CRF13_cpx (A, CRF01, G, J, U)	S. Arabia	96CM-1849	Cameroon	AF460972	94
60	04BD064	M	45	VCT	CRF14_BG	NA	X397	Spain	AF423756	92
61	03BD012	M	30	VCT	CRF15_01.B	Non migrant	06MMYKLD46	Malaysia	EF495062	95
62	03BD017 *	F	27	VCT	CRF15_01.B	Malaysia	06MMYKLD46	Malaysia	EF495062	97
63	00BD002 *	M	28	MSM	CRF25_A,G,U	NA	J11451	S. Arabia	DQ375319	97

* Sequence included in the tree: ^1^ M, male; F, female; H, hijra (transgendered people); ^2^ VCT, voluntary counselling and testing unit; ^3^ Hijra, transgendered people; ^4^ MSM, males who have sex with males; ^5^ PWID, People who inject drug; ^6^ FSW, female sex workers; ^7^ STI, sexually transmitted infections; ^8^ NA, history not available; ^9^ Spouse of a migrant who visited UAE; ^10^ Spouse of migrant who visited Saudi Arabia; ^11^ spouse of migrant who visited Malaysia; ^12^ South Korea, Malaysia, Singapore, Thailand, and Vietnam; ^13^ Saudi Arabia, UAE, India, and Pakistan; ^14^ Saudi Arabia and India; ^15^ Qatar and Saudi Arabia; ^16^ nt, nucleotide.

**Table 2:** HIV-1 subtype determination by *gag* and *env* region.

SL#	Specimen ID	Sex	Age (Years)	Source	Suptype (*gag)* p17/24	Suptype (*env)* C2/V3/C4
1	02BD054	Male	30	VCT	A1	A1
2	02BD040	Male	40	MSM	A1	A1
3	02BD066	Male	23	VCT	CRF01_AE	CRF01_AE
4	03BD075	Male	29	VCT	CRF01_AE	CRF01_AE
5	02BD047	Female	18	FSW	CRF01_AE	CRF01_AE

## Data Availability

The text of the article contains references to the nucleotide sequences of HIV-1 deposited in the Genebank, which are the main primary results of this study. The nucleotide sequences of HIV in the Genebank are publicly available.
